# Editorial: Mechanisms Underpinning the Link between Emotion, Physical Health, and Longevity

**DOI:** 10.3389/fpsyg.2017.01338

**Published:** 2017-08-02

**Authors:** Andrew H. Kemp

**Affiliations:** ^1^Department of Psychology and the Health and Wellbeing Academy, College of Human and Health Sciences, Swansea University Swansea, United Kingdom; ^2^School of Psychology and Discipline of Psychiatry, University of Sydney Sydney, NSW, Australia

**Keywords:** emotion, health, wellbeing, morbidity and mortality, longevity, stress, vagal function, GENIAL model

The study of emotion has been likened to the 100 Years War between England and France (Lindquist et al., 2013), a conflict that may relate, in part, to intense research interest on the brain, largely side-lining the body as a passive observer. However, research on the link between the brain and body may help to develop a more informed basis on which to understand our emotions. Such research also has important implications for premature morbidity and mortality. Each article published in this special issue and research topic is now briefly described, focusing on the main findings and relevance for better understanding the links between emotion, physical health, and longevity.

In the first article of this issue, Kemp et al. report on associations between common mental disorders and coronary heart disease (CHD), conditions that impose considerable burden on society. In their large epidemiological cohort study on the Brazilian population, participants with psychiatric comorbidity display a threefold increase in CHD, while a 1.5- to 2-fold increase was observed for non-comorbid conditions. Also in the issue, the same authors demonstrate that tricyclic medications—a class of antidepressants freely dispensed in Brazilian public health pharmacies—are associated with a twofold increase in CHD, relative to non-use. These findings highlight an urgent need for better understanding how extreme negative emotions—and their treatments—might impact on physical health over the longer term, just as other studies discussed below point to the need for greater insight into how positive health behaviors more commonly associated with physical wellbeing (e.g., exercise, diet) can improve emotional and mental health.

The biopsychosocial pathway to premature morbidity and mortality is a complex one, involving a host of closely intertwined mechanisms (Kemp and Quintana, 2013; Kemp et al., 2016b, in press), some of which are investigated in contributions to this special issue. One of these mechanisms includes ongoing unconscious emotion processing, which may contribute to adverse health outcomes. In this regard, a study in the issue by van der Ploeg et al. examines the validity of the Implicit Positive and Negative Affect Test (IPANAT; Quirin et al., 2009; Brosschot et al., 2014) and investigates whether changes in unconscious emotions (as measured by the IPANAT) relate to changes in a variety of physiological parameters. The IPANAT requires participants to rate six artificial words (vikes, tunba, ronpe, belni, sukov, safme) on six emotional adjectives (happy, cheerful, energetic, helpless, tense, inhibited) using a six-point Likert scale. Higher implicit negative affect was associated with higher systolic blood pressure, lower heart rate variability (HRV) and total peripheral resistance, and slower recovery of diastolic blood pressure. Also in the issue by the same research group, (Mossink et al.) subjective stress, worry and affect were measured over a 24-h period in addition to cortisol levels in saliva.

Implicit negativity bias was associated with increased cortisol levels, and higher levels of implicit sadness were associated with a stronger cortisol rise the next day. Research has seldom explored the physiological impacts of unconscious stress and further research in this area is needed.

A commonly measured variable in studies on stress is HRV. Unfortunately, researchers typically consider HRV as an epiphenomenon of the stress response (e.g., Friedman and Kern, 2014), rather than—as we would contend—a marker of vagal function responsible for a host of psychological and physiological processes that influence social ties and subsequent health and wellbeing (Kemp et al., in press). Also in the issue, a study by Porges et al. reports that individuals with lower vagal function respond with greater increases in salivary testosterone in response to observed violence. This finding was interpreted in line with a hierarchical model of autonomic activity (polyvagal theory; Porges, 2011) such that those with lower vagal function may be more vulnerable to environmental threat, increasing the potential for interpersonal conflict. The authors suggest that individuals with lower vagal function may have difficulties with emotional regulation, a suggestion that is supported by another article in the issue by Williams et al.

Reduced vagal function is also commonly reported in patients with a variety of psychiatric disorders, especially major depression (Kemp et al., 2010; Brunoni et al., 2013) and generalized anxiety disorder (Chalmers et al., 2014; Kemp et al., 2014). However, contradictory findings have also been reported, motivating another study in the issue by Kemp et al. Patients with melancholic depression were found to display increased heart rate and lower resting-state HRV relative to non-depressed controls. One explanation for the contradictory findings in the literature is that past studies have focused on major depressive disorder, rather than more homogeneous subtypes of depression such as melancholia. Critically, over the longer term these cardiac alterations may contribute to increased risk for premature morbidity and mortality. This possibility is supported by a recent meta-analysis on 21,988 participants without known cardiovascular disease (Hillebrand et al., 2013), reporting that vagal impairment predicts adverse cardiovascular events up to 15 years later.

Together, these findings have important implications for future research in this area, although it is also worth reminding newcomers about some of the methodological challenges, a motivation for including the paper by Quintana and Heathers in the issue.

Another area of research that has attracted considerable attention is the extent to which people are able to perceive and regulate visceral information from the body, a skill known as interoceptive ability. Interoceptive signals are communicated by the vagus nerve and a dedicated lamina-1 spinothalamocortical pathway to interoceptive centers in the brain (Craig, 2002, 2014; Critchley et al., 2004; Kemp et al., 2015). Atypical interoceptive sensitivity has been associated with psychopathology in adolescence and decreased socio-emotional competence in adulthood (Murphy et al., 2017). Another article in the issue by Jones et al. examined neural substrates for the volitional regulation of heart rate. Participants were instructed to either increase or decrease the level of an on-screen thermometer reflecting heart rate by either volitionally increasing or decreasing heart rate, respectively. Results demonstrate that heart rate during the arousal condition (76 bpm) significantly differed from the relaxation condition (72 bpm) indicating that instructions to participants had their desired effect. Decreases in heart rate were associated with activation of ventrolateral prefrontal and parietal cortices, regions known to be involved in cognitive reappraisal, and selective attention, respectively. Interestingly, participants could increase heart rate without any corresponding change in respiration, which may in part explain the relatively small observed changes in heart rate. Heart rate change also negatively correlated with anxiety scores. While no participant scored above the clinical threshold for anxiety, participants with more anxiety symptoms were less able to regulate their heart rate. Heart rate biofeedback training with a focus on prolonging the out-breath may help to diminish anxiety symptoms (Wells et al., 2012) and have a place in the management of anxiety disorders.

The study by Mallorquí-Bagué et al. in the issue examines the link between anxiety, affective reactivity and interoception in otherwise healthy people with joint hypermobility, an inherited condition associated with the expression of a common variation in the connective tissue protein, collagen. Hypermobility was assessed by asking participants whether they could touch the floor with their hands without bending their knees. Interestingly, joint hypermobility is associated with increased risk of anxiety and related disorders (Sanches et al., 2012). Interoception was found to mediate a positive association between joint hypermobility and state anxiety such that hypermobility is associated with heightened interoceptive sensitivity, which then contributes to state anxiety. Also reported was greater activation in the hypermobile group relative to controls during processing of sad vs. neutral images within a discrete set of brain regions associated with emotional processing. These findings highlight the dependence of emotional state on bodily context and further our understanding of how vulnerability to anxiety disorder might arise.

In another article of this issue, the study by Couto et al. examined two rare patients with respective damage of focal lesions to the insular cortex and to its subcortical tracts. Internally generated interoceptive streams were assessed through a heartbeat detection task, while externally triggered streams were examined via taste, smell, and pain recognition tasks. The patient with a lesion to the insula cortex displayed impaired internal signal processing while the patient with the subcortical lesion exhibited external perception deficits. The authors argue that the subcortical lesion may disrupt integrative contextual processing—spared in the patient with focal insula lesion—via a fronto-insulo-temporal network including the insula cortex as a critical hub. Importantly, this distinctive pattern was replicated when comparing patients' performance to that of subjects with lesions in other regions. This helps to confirm that the observed pattern of deficits relates to specific lesions rather than to nonspecific brain damage. These results point to a stratification of multimodal bodily signals that subsequently contribute to emotional awareness.

Considering evidence that antidepressant medications are not as safe as they were once believed (Licht et al., 2010; Kemp et al., 2014, 2016a), it is likely that positive health behaviors will play an increasingly important role in the management of mental health conditions. In their opinion article, Dash et al. make a strong argument in the issue for greater recognition that diet is a risk factor for common mental disorders and for public health strategies focused on dietary improvement. They argue that the physical health of patients should be given equal priority as a treatment target when considering patient mental health, and highlight the importance of a diet characterized by fruits, vegetables, whole grains, nuts, seeds, and fish while limiting intake of processed foods (Opie et al., 2015; see also: Jacka, 2017). Also in the issue, the study by Grung et al. focuses on the beneficial physiological effects of a fish diet on psychiatric inpatients including improved HRV, which are indices of emotional regulation as well as physical health. Two other papers in the issue focus on the effects of physical activity in bipolar disorder (Thomson et al.) and traumatic brain injury (TBI; Rzezak et al.). Also in the issue, the study by Garland et al. demonstrates that mindfulness may help to maintain trait positive affect and momentary positive cognitions in an upward spiral. Together, these articles highlight the utility of non-pharmacological options for improving health and wellbeing and provide some guidance on underlying mechanisms. Further research is needed to better inform and underpin the development of non-pharmacological interventions for mental-health conditions.

In the final study of the issue, IJzerman et al. suggest how relationship therapy might be modernized, a proposal that builds on Social Thermoregulation Theory (IJzerman et al., 2015). According to this theory, humans may adapt social behaviors and cognitions to temperature changes. For example, the authors describe how emotions like anxiety and sadness are associated with lower peripheral temperature, and that people respond to others' sadness with an increase in temperature. The authors explain that thermoregulation is crucial for survival and that individual differences in the need for thermoregulation may even predict attachment styles, health, and wellbeing. While the research in this area is still in its early stages, the links to social ties and health outcomes is intriguing, and is supported by an increasing body of evidence that was recently captured by the GENIAL model (Genomics—Environment—vagus Nerve—social Interaction—Allostatic regulation—Longevity; Kemp et al., in press).

In conclusion, all papers included in the special issue provide new insights into the pathways linking emotion, physical health, and longevity. Future research in this area would benefit from moving beyond the disciplinary dilemma, initiating multi-disciplinary exchange and facilitating new lines of interdisciplinary enquiry to better understand the complex pathways from emotion to longevity. The GENIAL model (Kemp et al., in press) is a first attempt to do exactly this and provides a foundation on which future research could be based.

## Author contributions

The author confirms being the sole contributor of this work and approved it for publication.

### Conflict of interest statement

The author declares that the research was conducted in the absence of any commercial or financial relationships that could be construed as a potential conflict of interest.
